# Patient-derived cells from recurrent tumors that model the evolution of *IDH*-mutant glioma

**DOI:** 10.1093/noajnl/vdaa088

**Published:** 2020-07-16

**Authors:** Lindsey E Jones, Stephanie Hilz, Matthew R Grimmer, Tali Mazor, Chloé Najac, Joydeep Mukherjee, Andrew McKinney, Tracy Chow, Russell O Pieper, Sabrina M Ronen, Susan M Chang, Joanna J Phillips, Joseph F Costello

**Affiliations:** 1 Department of Neurological Surgery, University of California, San Francisco, California, USA; 2 Biomedical Sciences Graduate Program, University of California, San Francisco, California, USA; 3 Department of Radiology and Biomedical Imaging, University of California, San Francisco, California, USA; 4 Department of Biochemistry and Biophysics, University of California, San Francisco, California, USA

**Keywords:** hypermutation, *IDH1*-mutant glioma, intracranial xenograft, intratumoral heterogeneity and evolution, patient-derived cells

## Abstract

**Background:**

*IDH-*mutant lower-grade gliomas (LGGs) evolve under the selective pressure of therapy, but well-characterized patient-derived cells (PDCs) modeling evolutionary stages are lacking. *IDH*-mutant LGGs may develop therapeutic resistance associated with chemotherapy-driven hypermutation and malignant progression. The aim of this study was to establish and characterize PDCs, single-cell-derived PDCs (scPDCs), and xenografts (PDX) of *IDH1*-mutant recurrences representing distinct stages of tumor evolution.

**Methods:**

We derived and validated cell cultures from *IDH1-*mutant recurrences of astrocytoma and oligodendroglioma. We used exome sequencing and phylogenetic reconstruction to examine the evolutionary stage represented by PDCs, scPDCs, and PDX relative to corresponding spatiotemporal tumor tissue and germline DNA. PDCs were also characterized for growth and tumor immortality phenotypes, and PDX were examined histologically.

**Results:**

The integrated astrocytoma phylogeny revealed 2 independent founder clonal expansions of hypermutated (HM) cells in tumor tissue that are faithfully represented by independent PDCs. The oligodendroglioma phylogeny showed more than 4000 temozolomide-associated mutations shared among tumor samples, PDCs, scPDCs, and PDX, suggesting a shared monoclonal origin. The PDCs from both subtypes exhibited hallmarks of tumorigenesis, retention of subtype-defining genomic features, production of 2-hydroxyglutarate, and subtype-specific telomere maintenance mechanisms that confer tumor cell immortality. The oligodendroglioma PDCs formed infiltrative intracranial tumors with characteristic histology.

**Conclusions:**

These PDCs, scPDCs, and PDX are unique and versatile community resources that model the heterogeneous clonal origins and functions of recurrent *IDH1*-mutant LGGs. The integrated phylogenies advance our knowledge of the complex evolution and immense mutational load of *IDH1*-mutant HM glioma.

**Key Points**

- A unique resource to model intratumoral heterogeneity and evolution.
- Integrated phylogenies that reveal patterns of chemotherapy-induced hypermutation.

**Importance of the Study**

Intratumoral heterogeneity and tumor evolution contribute to therapeutic failure, but patient-derived cells (PDCs) that model these critical features are lacking. PDCs of *IDH*-mutant gliomas are particularly poorly represented, and few if any are widely used in neuro-oncology research. We established robust, versatile, and well-characterized PDCs of an *IDH1*-mutant astrocytoma and an *IDH1*-mutant oligodendroglioma that represent defined evolutionary stages of chemotherapy-induced hypermutation. The PDCs retain tumor subtype-defining features over time as well as classic hallmarks of cancer, including anchorage-independent growth and cell immortality. The integrated phylogenies composed of PDCs, single-cell-derived PDCs, patient-derived xenografts, and corresponding spatiotemporal tumor tissue samples also provide new insight into the clonality, evolutionary pattern, and immense mutational load of hypermutated *IDH*-mutant gliomas.

*IDH*-mutant lower-grade gliomas (LGGs) are relatively slow-growing, infiltrative tumors that typically occur in the cerebrum of adults. They are classified histologically and molecularly as oligodendroglioma harboring mutation of *IDH1* or *IDH2* (*IDH*-mutant) and codeletion of chromosomal 1p and 19q arms, or astrocytoma harboring *IDH* mutation often accompanied by *ATRX* loss and *TP53* mutation.^1,2^ Typically, *IDH*-mutant LGGs are treated with surgical resection followed by chemotherapy,^3^ though patients considered high risk with an age older than 40 or subtotal resection or biopsy only may receive chemo-radiotherapy,^4,5^ most commonly with temozolomide (TMZ). Despite the clinical intervention, gliomas recur.^6^ The time to recurrence is highly variable, and some *IDH*-mutant LGGs may recur as a low-grade tumor, while others undergo malignant progression and recur as a higher-grade tumor.^7^

At recurrence, *IDH-*mutant LGGs may delete the mutant *IDH* allele or evolve into hypermutated (HM) glioma after TMZ treatment.^8–16^ TMZ-associated hypermutation in *IDH*-mutant LGGs and glioblastoma is characterized by an increased mutational burden dominated by C:G>A:T nucleotide transitions and mismatch repair (MMR) deficiency.^17–20^ HM *IDH*-mutant LGGs are further characterized by mutations in key genes of the PI3K–AKT–mTOR signaling pathway.^8^ Clinical trials have been designed to begin to address the treatment of HM glioma, highlighting the need for preclinical models to study this genetically distinct recurrence. As with primary *IDH*-mutant LGGs, HM recurrences evolve and exhibit intratumoral heterogeneity. Evolution and intratumoral heterogeneity may underlie therapeutic failures, but widely used glioma cell lines do not model these key features.

Well-characterized in vitro and in vivo models are needed to study *IDH*-mutant LGGs, particularly after they have evolved under the strong selective pressure of chemotherapy.^21,22^ Kelly et al.^23^ established a 1p/19q codeleted cell line, BT-088, that is *IDH* wildtype and resistant to chemotherapy. Yip et al.^2^ performed tumor-only whole-genome sequencing on BT-088, demonstrating hypermutation. Wakimoto et al.^16^ established 7 xenografts with heterozygous *IDH1* mutation that maintained 2-HG production through serial xenografting. Of these 7 *IDH*-mutant xenografts, 3 were treated with TMZ and 2 acquired tertiary mutations, though it is unclear if they are HM. Several of these patient-derived cells (PDCs) have been well characterized metabolically.^24,25^ In most cases, a lack of corresponding tumor tissue makes it difficult to distinguish mutations arising in vivo versus in vitro. Similarly, tumor-only sequencing hampers distinguishing somatic mutations from normal genetic variation. Furthermore, in nearly every widely used human glioma cell model, the corresponding stage of tumor evolution is not known. In this study, we describe a robustly characterized PDC resource not limited by these factors. Through the integrated phylogenetic analysis, this study also offers new insight into the dramatic evolution of HM glioma.

## Methods

### Sample Acquisition

Fresh surgical specimens were acquired from patients undergoing surgical resection for recurrent glioma after TMZ treatment by the Neurological Surgery Brain Tumor Center at the University of California, San Francisco (UCSF). The research was approved by the Institutional Review Board at UCSF and sample use was approved by the Committee on Human Research at UCSF. Informed consent was obtained from all patients.

### Patient-Derived Cells

Tumor tissue was dissociated with papain (Worthington) for 30 minutes. The suspension was passed through a 70 μM cell strainer, twice, then a 40 μM cell strainer, twice, to achieve a single-cell suspension. To maximize the number and diversity of successful cultures, dissociated cells were placed into 2 media conditions known to support glioma cell growth: (1) serum-free, glioma neural stem (GNS) cell medium,^26–28^ supplemented with EGF (animal-free; PeproTech), bFGF (animal-free; PeproTech), and PDGF-AA (animal-free; PeproTech) or (2) 10% fetal bovine serum (FBS). GNS media comprises of Neurocult NS-A (Stem Cell Technologies) supplemented with N2 (Invitrogen), B27 (without vitamin A; Invitrogen), 100 μg/mL streptomycin and 100 units/mL penicillin “G” (UCSF Cell Culture Facility), 2 mM l-glutamine (UCSF Cell Culture Facility), and 0.1 mg/mL sodium pyruvate (UCSF Cell Culture Facility). 10% FBS media comprises of DMEM/Ham’s F-12 1:1 Mix (UCSF Cell Culture Facility) supplemented with 10% FBS (Hyclone, characterized) and 100 μg/mL streptomycin and 100 units/mL penicillin “G.” PDCs were grown in a humidified environment at 37°C with 5% CO_2_. The passage (P) number of the PDC is denoted in each experiment. PDCs were determined to be mycoplasma free by testing with the MycoAlert PLUS kit (Lonza).

### Single-Cell Subclones of PDCs

We derived clones of the astrocytoma SF10602 PDC GNS and the oligodendroglioma SF10417 PDC GNS by sparsely seeding 500 cells into laminin-coated 15 cm tissue culture dishes. Colonies formed from single cells were isolated with cloning cylinders and Accutase (Innovative Cell Technologies). Seventeen clones were derived from SF10602 PDC GNS and 31 from SF10417 PDC GNS. Of the 48 clones, which were all *IDH1*-mutant by Sanger sequencing, we selected 5 SF10602 PDC GNS and 4 SF10417 PDC GNS clones for exome sequencing.

### DNA Isolation

Cells were detached from culture flasks with Accutase, pelleted, and washed with phosphate-buffered saline before pelleting and snap-freezing. DNA was extracted and cleaned from thawed cell pellets and snap-frozen tissue with phenol, chloroform, and isoamyl alcohol as previously described.^8^ DNA was resuspended in Tris-EDTA (TE) (Teknova) and stored at 4°C.

### Exome Sequencing and Mutation Calling

Whole-exome capture was performed with SeqCap EZ Exome V3 (Nimblegen) and sequenced on HiSeq 2500 or HiSeq 4000 instrumentation (Illumina). Genomic alignment was performed and mutations were called against normal samples as previously described.^8^ Raw data can be accessed through the European Genome-Phenome Archive at accession number EGAS00001003992.

### Copy Number Analysis

The copy number across chromosomes was estimated using Parent-Specific Circular Binary Segmentation.^29,30^

### Construction of Tumor Phylogenies

Tumor phylogenies were constructed using ordinary least-squares minimum evolution from a distance matrix of Manhattan distances, as previously described.^31^

### Mutational Signature Analysis

Missense mutations called from whole-exome sequencing for each sample were used as input for the mutational signature-calling tool, deconstructSigs.^32^ We quantified the resulting mutational signatures by their proportional contribution.^33^

### *IDH1* and *TERT* Promoter Sanger Sequencing

*IDH1* status was validated by PCR with a 2X Phusion High-Fidelity master mix (New England Biolabs [NEB]) and Sanger sequencing (Quintara Biosciences), as previously described.^8^*TERT* promoter (*TERT*p) mutational status was tested as previously described.^34^ Part of the *TERT*p encompassing the common mutations 124 and 146 base pairs upstream of the transcription start site was amplified by PCR with a GC-Rich PCR System (Roche) and Sanger sequenced. All PCR product sequences were aligned to a reference sequence using Sequencher (Gene Codes).

### Measurement of 2-HG

Metabolites were extracted from 1.5–2.5 × 10^7^ cells using the dual-phase extraction method, magnetic resonance spectra acquired, and spectral processing was done as previously described.^35^

### Colony Formation Assays

Growth in soft agar is a well-established phenotype of transformed cells.^36^ About 1000 cells were seeded into 0.35% (w/v) ultra-low melting point agarose (Invitrogen) in GNS or FBS media between layers of 0.7% ultra-low melting point agarose in 6-well plates.^37^ After 4–5 weeks, colonies were stained with 0.005% crystal violet (Sigma) and counted under a microscope.

### C-circle Assay

A C-circle assay was performed as previously described^38^ on DNA that was stored in TE at 4°C. Briefly, DNA was digested with *Rsa*I and *Hinf*I (NEB) and amplified with Φ29 DNA polymerase. DNA was dotted onto a nylon membrane, cross-linked, and labeled with^32^P-(CCCTAA)_3_.

### Telomere Restriction Fragment Length Analysis

Telomere restriction fragment length analysis was done as previously described.^39^ Purified genomic DNA, isolated with phenol/chloroform/isoamyl alcohol (SF10602 PDC GNS, SF10602 PDC FBS, SF10417 PDC GNS) or a DNeasy Blood and Tissue kit (Qiagen; UMUC3 and U2OS), was digested with *Hinf*I, *Alu*I, *Hae*III, *Rsa*I, *Hha*I, and *Msp*I (NEB) and then resolved on an agarose gel. The gel was denatured, dried, and prehybridized. Telomeres were visualized on a Phosphorimager screen after hybridization to a ^32^P-(CCCTAA)_3_ probe.

### Luciferase-Modified PDCs

SF10417 PDC GNS and SF10602 PDC GNS were modified to stably express luciferase for use in in vivo bioluminescent imaging by infection with Firefly Luciferase Lentifect Purified Lentiviral Particles (Genecopoeia) at an multiplicity of infection of 7. Cells were exposed to 150 μg/mL luciferin (d-luciferin; Gold Biotechnology) and imaged on an IVIS Spectrum (Perkin Elmer) to confirm stable expression.

### Intracranial Patient-Derived Xenografts

Animal experiments were performed to comply with the current laws of the country, and the UCSF Institutional Animal Care and Use Committee approved all animal protocols (IACUC protocol AN111064-03B to Dr. Theodore Nicolaides, and IACUC protocol AN175997-01 to Dr. Tomoko Ozawa at UCSF). All animals were housed under aseptic conditions and had access to food and water ad libidum.

Initial xenografts were established in 5-week-old female athymic mice (Simonsen). Mice were anesthetized with 100 mg/kg ketamine and 10 mg/kg xylazine, an incision made in the scalp, and a hole made in the skull with a 25G needle, through which 300 000 luciferase-modified cells were injected into the caudate putamen, as previously described.^40^ Second passage patient-derived xenografts (PDX), which were derived from the cells cultured from the initial PDX, were established in 5-week-old female athymic mice (Harlan). Mice were anesthetized with 2–3% isofluorane, an incision made in the scalp, and a hole made with a burr drill through which 300 000 cells were delivered stereotactically to 1.5 mm anterior and 1.5 mm lateral of bregma, and 2.5 mm deep.

Mice were monitored daily until they reached a moribund state or demonstrated 15% weight loss, at which point they were euthanized with CO_2_ inhalation followed by cervical dislocation, and brains were immediately removed.

### Tissue Processing and Analysis

After euthanasia, brains were extracted, divided into pieces and (1) placed into GNS media for dissociation into the culture, (2) flash-frozen for DNA isolation, and/or (3) fixed overnight in 10% neutral buffered formalin. Fixed tissue was embedded in paraffin and sectioned. H&E staining was performed according to routine procedures. Immunohistochemistry (IHC) was performed using a VENTANA BenchMark XT (Roche) or VENTANA Discovery Ultra by the UCSF Brain Tumor Center Tissue Core using anti-IDH1 R132H mouse monoclonal antibody (Histobiotec, DIA-H09) as described previously.^12^

## Results

### Patient Characteristics and Clinical Histories

**Figure 1.**
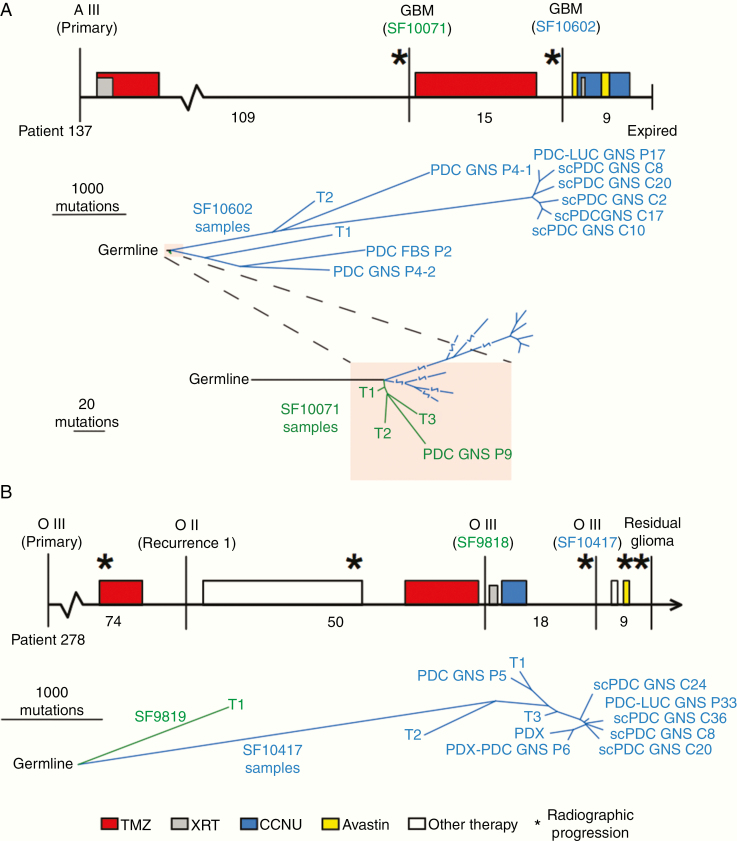
Phylogenies of *IDH*-mutant astrocytoma and oligodendroglioma PDCs, scPDCs, and PDX integrated with spatiotemporal tumor tissue samples. (A, *upper panel*): The clinical timeline of astrocytoma Patient 137, drawn to scale. Vertical lines indicate surgical resection; stars represent radiographic progression; filled rectangles indicate treatment period and fill color indicates treatment type (see key at bottom). Months between surgical resections are provided below the timeline. The tumor classification is listed above each surgical time point, and surgeries from which samples were sequenced are designated with an SF number. *Lower panel*: A tumor phylogeny was constructed from single-nucleotide somatic mutations. Line length is proportional to the number of mutations. *Lowest panel*: A zoomed-in view of the shaded portion of the tree showing the relationship between samples of the non-HM first tumor recurrence (SF10071) and the HM samples of the second recurrence (SF10602). (B, *upper panel*): The clinical timeline of oligodendroglioma Patient 278. *Lower panel*: A tumor phylogeny of tumor tissue and PDCs of Patient 278. FBS, cells cultured in media containing fetal bovine serum; GNS, cells cultured in glioma neural stem cell media; PDCs, patient-derived cells; scPDCs, single-cell-derived patient-derived cells; PDC-LUC, luciferase-modified PDC; T1, T2, T3, spatially distinct pieces of tumor tissue; PDX, patient-derived intracranial xenograft; PDX-PDC, PDC derived from PDX; A, astrocytoma; O, oligodendroglioma; TMZ, temozolomide; XRT, radiation therapy; CCNU, lomustine; P#, passage number.

Tumors change significantly over time, but most widely used cell lines are derived from newly diagnosed tumors. For *IDH1*-mutant LGGs in particular, there are few cell lines available that span multiple stages of tumor evolution. In order to develop models of recurrent *IDH-*mutant tumors, we cultured fresh tumor tissue at recurrence from 2 patients with prior TMZ treatment who were originally diagnosed with *IDH1*-mutant LGG. The clinical timelines include treatment history, progression events, surgical resections, and tumor grading (Figure 1A and B, upper panel). We acquired tissue from the second recurrence of a female patient (Patient 137) originally diagnosed with a grade III astrocytoma. The patient received TMZ after surgical resection of the primary tumor and again after surgical resection of the first recurrence (Figure 1A). Tissue was acquired from the third recurrence of a male patient (Patient 278) initially diagnosed with a grade II oligodendroglioma. This patient received TMZ prior to resection of the first recurrence and prior to resection of the second recurrence (Figure 1B). Patient-derived tumor cells were established in GNS and FBS culture media from the recurrent astrocytoma, while the recurrent oligodendroglioma yielded GNS cultures and a PDX. For both patients, we also established single-cell-derived PDCs (scPDCs).

### Faithful Cell Models of an HM Astrocytoma (Patient 137) and an HM Oligodendroglioma (Patient 278)

It is not clear if many of the widely used glioblastoma cell lines model their original tumor due to a lack of genetic characterization of their originating tumor tissue and due to the lack of a corresponding normal germline DNA to distinguish the somatic mutations from normal genetic variants. To overcome these limitations for our *IDH1*-mutant gliomas, we performed exome sequencing on PDCs, scPDCs, and PDX samples along with multiple tumor tissue samples and germline normal DNA, for a total of 28 exomes across the 2 patients (Supplementary Table 1). Through integrated phylogenetic analyses, we observed that all 15 samples from the astrocytoma derived from an inferred common ancestor defined by 41 mutations including *IDH1*, *ATRX*, and *TP53* (Supplementary Table 2). The overall structure of the phylogenetic tree reveals 2 major branch points that define the relative timing and divergence of large clonal expansions (Figure 1A, lower panel). One major branch point distinguishes samples of the first non-HM recurrence (SF10071) from the second recurrence (SF10602), which acquired TMZ-associated hypermutation. The second major evolutionary branch point occurs within samples from the second recurrence. The 3 samples on the lower HM branch share a common ancestor composed of 468 mutations, while the 8 samples in the upper branch have an entirely different nearest common ancestor defined by 970 shared mutations. From this analysis, we infer the presence of at least 2 independent founder hypermutation events within this astrocytoma.

Passage 4 (P4) of the PDC GNS from the second recurrence contains an admixture of 2 independent HM clonal expansions based on presence of the 970 shared mutations on the upper branch along with 1016 mutations that are also in samples on the lower branch in common with PDC FBS P2 from the second recurrence. The mutations within this PDC, therefore, were partitioned computationally into 2 groups on the phylogenetic tree, P4-1 and P4-2. A subclone emerging from the inferred HM clone P4-1 evolved into a dominant clone by P17 (SF10602 PDC-LUC GNS P17). Thus, the inter- and intra-PDC heterogeneity faithfully model intratumoral heterogeneity in this patient.

To begin to investigate intra-PDC heterogeneity at single-cell resolution, scPDCs were derived and expanded from P21. All scPDCs maintained heterozygous *IDH1* R132H (Supplementary Figure 1). Of 20 scPDCs that continued to expand, 5 were selected for exome sequencing. In contrast to the large differences in mutations between passages, the scPDCs are more closely related, although there is a mean of 132 unique mutations per scPDC. An additional *IDH1*-mutant but non-HM PDC (SF10071 GNS P9) was derived from the first recurrence of this patient. Exome sequencing demonstrated that this non-HM PDC shared a proportion of the mutations seen in 3 spatially distinct tumor tissue samples (T1, T2, T3) as well as the 41 truncal mutations found in all samples from the first and second recurrence of this patient. Taken together, we infer that the astrocytoma PDCs and scPDCs represent multiple stages of tumor evolution, including the pre-hypermutation state and 2 distinct founder HM clonal expansions. The collection also represents HM clones with substantial additional mutagenesis superimposed on the founder HM clones, providing further insights into the complex evolution and substantial mutagenesis likely occurring during the TMZ treatment period.

An oligodendroglioma PDC from recurrence 3, SF10417 PDC GNS P5, also exhibited TMZ-associated hypermutation, harboring 5692 mutations, 99% of which were shared by tumor tissue sample T1 (Figure 1B, lower panel). Furthermore, 3967 mutations in this PDC are shared with all 3 spatially distinct HM tissue samples of the tumor (T1, T2, T3), suggesting this culture represents a relatively recent common ancestor. In tumor tissue from recurrence 2 (SF9818), we also detected hypermutation, which was composed of 1599 mutations representing an earlier HM clonal expansion that was not observed in recurrence 3.

We examined clonal heterogeneity further in samples from this oligodendroglioma by deriving luciferase-modified cells for xenografts from passage 33 (SF10417 PDC-LUC GNS P33) and scPDCs from passage 22 (P22) of SF10417 PDC GNS (Figure 1B, lower panel). Thirty-one scPDCs were derived, and all maintained heterozygous *IDH* R132H (Supplementary Figure 2). Exome sequencing was performed on 5 of the scPDCs. The evolutionary relationships of clones in the tumor tissue to those in corresponding cell cultures paralleled the observations in the astrocytoma patient. We observed close relationships among scPDCs and between scPDCs and P33 cells from this oligodendroglioma patient. Nevertheless, scPDCs still had an average of 171 unique mutations per clone. The luciferase-modified P33 line was injected intracranially into nude mice, and the resulting tumors (SF10417-PDX) shared a majority of mutations found in the tumor tissue and PDCs but had unique mutations as well. A PDC was derived from this first passage PDX. Exome sequencing at passage 6 (SF10417 PDX-PDC GNS P6) showed that the mutation profile is very similar to its PDX of origin, suggesting this versatile HM clone adapts to in vitro and in vivo growth conditions. All of the oligodendroglioma tumor samples shared driver-like mutations in *IDH1*, *TERT*p, *CIC*, and *FUBP1* (Supplementary Table 3). Loss of heterozygosity (LOH) was retained along the full length of 1p/19q chromosome arms in all PDCs, scPDCs, and PDX. However, multiple regions on the 1p/19q arms evolved to a copy neutral LOH state in the PDCs and PDX (Supplementary Figure 3).

To verify that the hypermutation in the astrocytoma and oligodendroglioma samples is TMZ-associated, all samples were evaluated for mutational signatures. Those with high mutation burden were found to be dominated by mutational signature 11, which is characterized by C:G>A:T nucleotide transitions and is associated with TMZ exposure (Supplementary Figure 4).^33^

### Faithful Modeling of Canonical Drivers and Cellular Phenotypes

**Figure 2.**
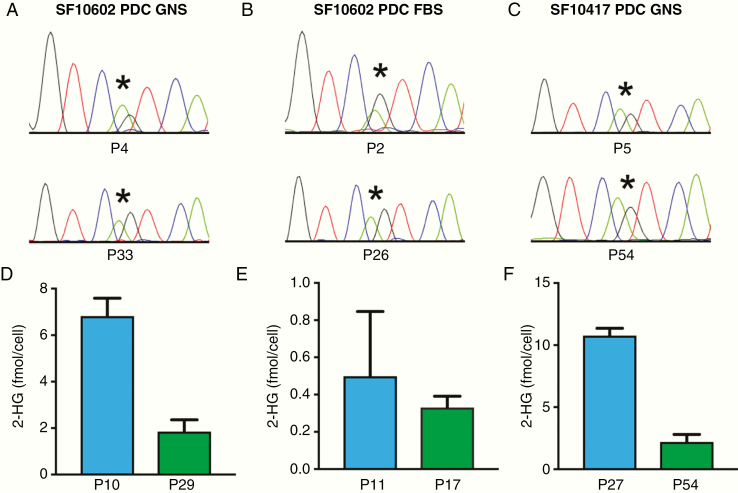
PDCs retain heterozygous *IDH1* R132H through serial passage and continue to produce 2-HG. (A–C) Each of the 3 PDCs retains *IDH1* R132H through multiple passages as determined by Sanger sequencing and (D–F) produces 2-HG through multiple passages, as measured by NMR. *The location of the heterozygous G>A mutation in *IDH1*.

Despite the substantial evolution of these tumors, the PDCs retained subtype-defining features. The PDCs from the astrocytoma second recurrence maintained heterozygous *IDH1* mutation through extensive passaging (Figure 2A and B), as did the oligodendroglioma recurrence 3 PDCs (Figure 2C). While 2-HG production decreased in later passages, it remained readily detectable (Figure 2D–F).

**Figure 3.**
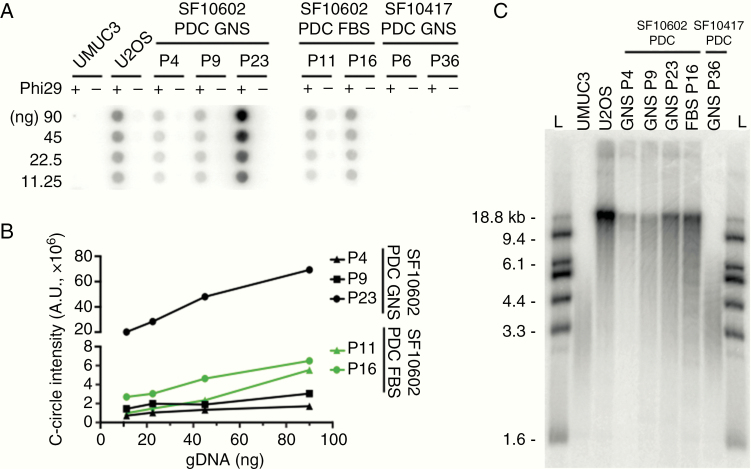
*ATRX*-mutant PDCs are positive for the ALT phenotype. (A) The *ATRX*-mutant SF10602 PDC GNS and PDC FBS cultures are positive for C-circle amplification across multiple passages. In contrast, the *ATRX* wildtype SF10417 GNS culture is negative for C-circles. A human bladder transitional cell carcinoma cell line (UMUC3) and a human osteosarcoma cell line (U2OS) serve as negative and positive controls for C-circle amplification, respectively. (B) Signal intensity was quantified in arbitrary units and plotted. (C) Telomere restriction fragment length analysis shows an accumulation of long chromosomes and a heterogeneous telomere length distribution characteristic of ALT. The ALT cells show a tighter and shorter telomere length distribution. *L*, DNA ladder.

Maintenance of telomere length underlies tumor immortality, a hallmark of human cancer. We examined PDC genotypes and phenotypes associated with telomere maintenance, which differ in astrocytoma and oligodendroglioma. Astrocytomas are typically characterized by loss-of-function mutations in *ATRX*, which are tightly associated with the alternative lengthening of telomeres (ALT) phenotype. The astrocytoma tumor tissue samples and PDCs all shared the missense ATRX mutation G1567D. Recurrence 2 PDCs were positive for partially single-stranded telomeric DNA circles, or C-circle amplification,^41^ a quantitative assay for ALT, with the quantity of C-circles increasing over time (Figure 3A and B). Conversely, the *ATRX*-intact and *TERT*p-mutant oligodendroglioma PDCs were negative for C-circle amplification (Figure 3A). The ALT-negative and *TERT*p-mutant oligodendroglioma PDCs had telomeres ranging from ~2 to 6 kb, consistent with maintenance of critically short telomeres, while ALT-positive astrocytoma PDCs had a more heterogeneous distribution of telomere lengths that included an accumulation of much longer telomeres at 18.8 kb (Figure 3C). Previously, we demonstrated in the oligodendroglioma PDCs that *TERT* expression is reduced upon GABPB1 inhibition.^42^ These recurrent astrocytoma and oligodendroglioma PDCs are therefore suitable for studying mechanisms underlying tumor cell immortality, including ALT and telomerase activation driven by *TERT*p-mutation.^43,44^

### PDCs Exhibit Tumorigenic Phenotypes In Vitro and In Vivo

**Figure 4.**
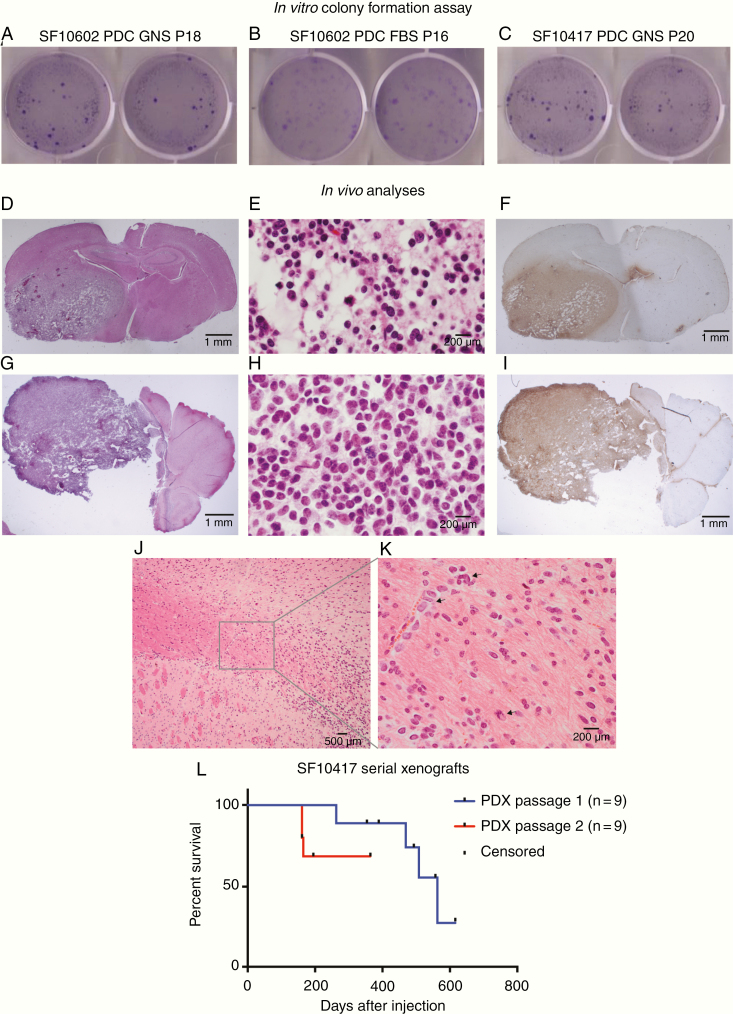
PDCs demonstrate tumorigenic properties in vitro and in vivo. To measure in vitro tumorigenesis, 1000 cells each of (A) astrocytoma SF10602 PDC GNS, (B) SF10602 PDC FBS, and (C) oligodendroglioma SF10417 PDC GNS were seeded into soft agar. After 4 weeks, they were stained with crystal violet and yielded an average of 185, 117, and 218 colonies, respectively. (D–L) To measure in vivo tumorigenesis, 300 000 cells were implanted into the right cerebrum of 5-week-old female athymic mice. (D) H&E staining of a coronal section of the mouse brain demonstrating the infiltrative nature of the tumor. (E) The tumor recapitulates oligodendroglioma histologic features and (F) is positive for IDH1 R132H by IHC. A cell line was established from this tumor and re-implanted in 5-week-old female athymic mice, where (G) it again formed infiltrative tumors that (H) recapitulate oligodendroglioma histology and (I) are positive for IDH1 R132H by IHC. (J) Tumor cells infiltrated from the site of injection in the striatum into and within the corpus callosum, (K) as single cells and as clusters of tumor cells (arrows) along blood vessels. (L) Upon the first xenotransplantation, SF10417 PDC GNS formed tumors in 4 of 9 mice over a protracted time period, consistent with the generally slower growth of this tumor subtype. Upon serial xenotransplantation, SF10417 PDX-PDC GNS formed tumors in 3 of 9 mice in a reduced time period. Tick marks indicate censored animals.

Anchorage-independent growth is another common feature of transformed cell lines, whereas nontransformed cells typically require adhesion to the extracellular matrix to survive and proliferate. We therefore used the soft-agar assay on the PDCs from the astrocytoma and oligodendroglioma. Both readily formed colonies in soft agar, suggesting these PDCs have retained this classic growth phenotype of transformed cells (Figure 4A–C).

To determine if the PDCs are versatile for in vivo studies, we examined the ability of both the astrocytoma and oligodendroglioma PDCs to form tumors in the brain of athymic mice. Although we followed the cohort implanted with the astrocytoma PDCs (SF10602 PDC-LUC GNS) for more than 600 days, including luminescence measurements, monitoring mouse weight, and neurologic status, we saw no evidence of tumor formation. The cause of the failure to produce tumors in vivo is unknown, but could be due to several factors, including but not limited to the particular genotype or cellular state of these tumor cells that may be incompatible with orthotopic in vivo growth. In contrast, luciferase-modified oligodendroglioma PDCs (SF10417 PDC-LUC GNS) yielded infiltrative tumors in 4 of 9 mice and the tumors exhibited oligodendroglioma histologic features (Figure 4D and E) and maintained IDH1 R132H expression as measured by IHC on formalin-fixed, paraffin-embedded xenograft tissue (Figure 4F). A PDC was derived from the initial PDX shown in Figure 4D–F and was re-implanted orthotopically into mice. Secondary xenografts formed in 3 of 9 mice that continued to exhibit oligodendroglioma histology, although with slightly less infiltration (Figure 4G and H), and continued to express IDH1 R132H (Figure 4I). Tumor cells migrated from the site of injection (Figure 4J), as single cells and clusters of cells, and along blood vessels (Figure 4K). Over the course of these serial xenografts, the time to endpoint was reduced to nearly one-third of the original time course (Figure 4L).

## Discussion

Tumor genomes change over time, particularly under the selective pressure of chemotherapy. However, few tumor cell models represent glioma at recurrence. Cells that model the spatially distinct intratumoral heterogeneity of the originating tumor and are traceable to specific evolutionary time points are also lacking. Furthermore, many glioma cell lines lack corresponding normal germline DNA sequencing, which limits the ability to distinguish somatic mutation from normal genetic variants. Here, we successfully derived and characterized PDCs and scPDCs that maintain diagnostic molecular features and key oncogenic drivers of *IDH1*-mutant astrocytoma and oligodendroglioma. These HM PDCs were derived from recurrent, post-TMZ tumors and show genetic alterations commonly associated with hypermutation,^8,45,46^ such as high mutation burden, C:G>A:T nucleotide transitions in MMR and PI3K pathway genes. The evidence of mutational signature 11 in the PDCs strongly suggests that the differences between samples are attributable to growth dynamics and selection during the TMZ treatment period. The majority of mutations and heterogeneity observed in these PDCs may therefore originate in the patient, though a proportion may be newly acquired or selected ex vivo. As these cultures propagate after dilution to single cells, individual cultures can be isolated and/or mixed at defined portions for clonal competition analyses, providing an opportunity to explore how different HM tumor cells compete in controlled environments. They may also be suitable for single-cell cloning after genetic editing.

In vitro models of glioma suggest the dependency on mutant IDH1 may be transient.^47^ Furthermore, during the evolution of *IDH1*-mutant tumors in patients, approximately 12% exhibit deletion or amplification of the *IDH* locus, decreased 2-HG, and increased proliferation, suggesting *IDH* mutation may be unnecessary at later stages of tumor evolution.^12–14,16,48–50^ In this regard, serial xenografts of our oligodendroglioma (SF10417 PDC GNS) provide an additional model to genetically engineer copy number changes of *IDH1* and specifically test for causality.^51^ Additionally, these PDCs have utility in the study of telomerase and ALT-mediated mechanisms of cellular immortality. Our PDCs continue to proliferate over extended passaging in vitro and have proven useful for drug testing and functional studies.^42–44,52^

## Supplementary Material

vdaa088_suppl_Supplementary_Figure_S1

vdaa088_suppl_Supplementary_Figure_S2

vdaa088_suppl_Supplementary_Figure_S3

vdaa088_suppl_Supplementary_Figure_S4

vdaa088_suppl_Supplementary_Tables

vdaa088_suppl_Supplementary_Legends

## Notes

**Authorship statement**. Conception and design: L.E.J. and J.F.C. Data collection: L.E.J., C.N., J.M., A.M., and T.C. Data analysis and interpretation: L.E.J., S.H., M.R.G., T.M., C.N., J.M., T.C., J.J.P., and J.F.C. Manuscript preparation: L.E.J., S.H., M.R.G., R.O.P., S.M.R., J.J.P., and J.F.C. Tumor sample procurement: J.J.P. and S.M.C.
