# Supplementary figures and images for: Patient-derived cells from recurrent tumors that model the evolution of IDH-mutant glioma
Source: Neurooncol Adv. 2020 Jul 16;2(1):vdaa088. doi: 10.1093/noajnl/vdaa088 (PMC7462278; doi:10.1093/noajnl/vdaa088)

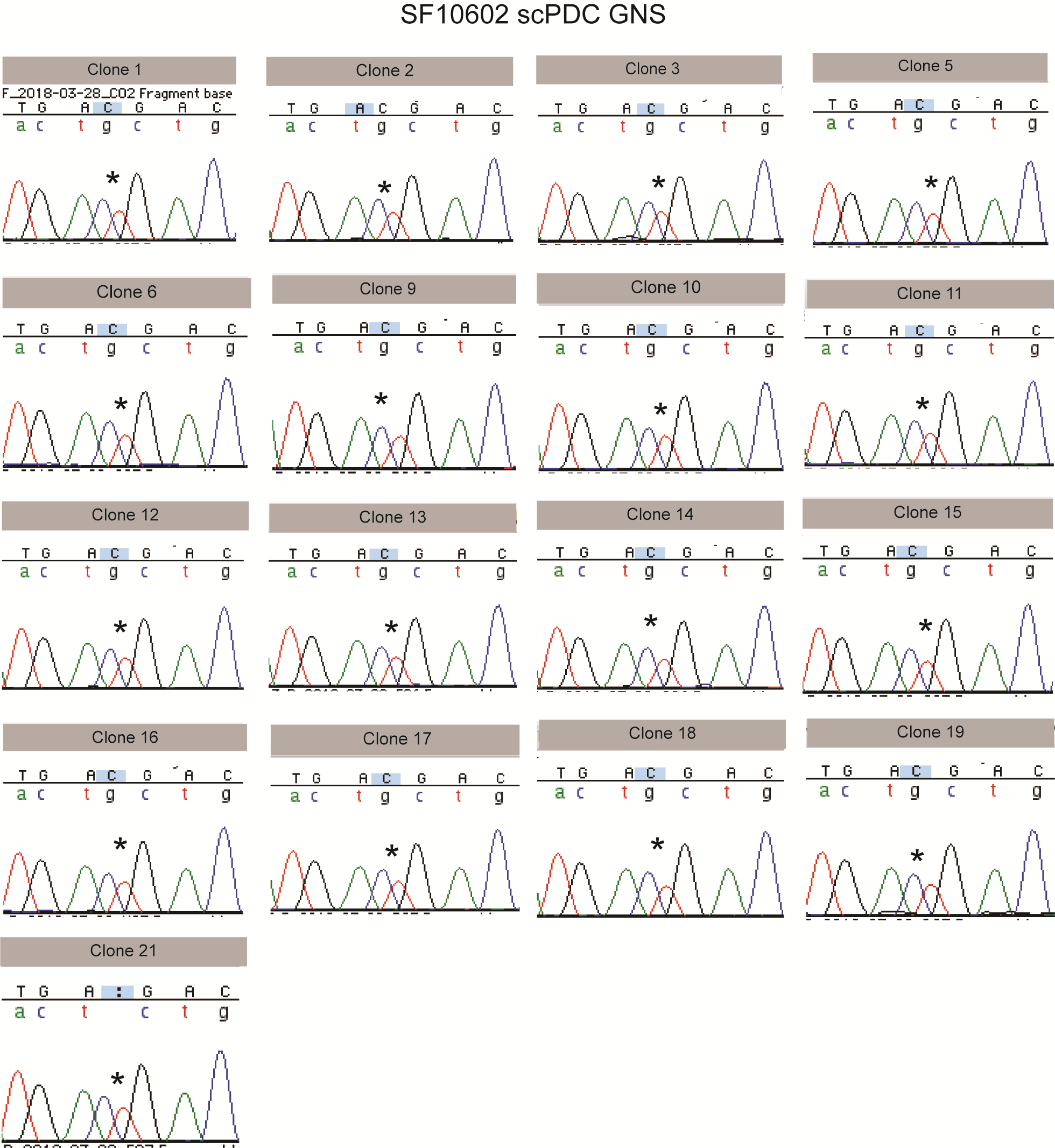

Supplement: vdaa088_suppl_Supplementary_Figure_S1 [file vdaa088_suppl_supplementary_figure_s1.png]

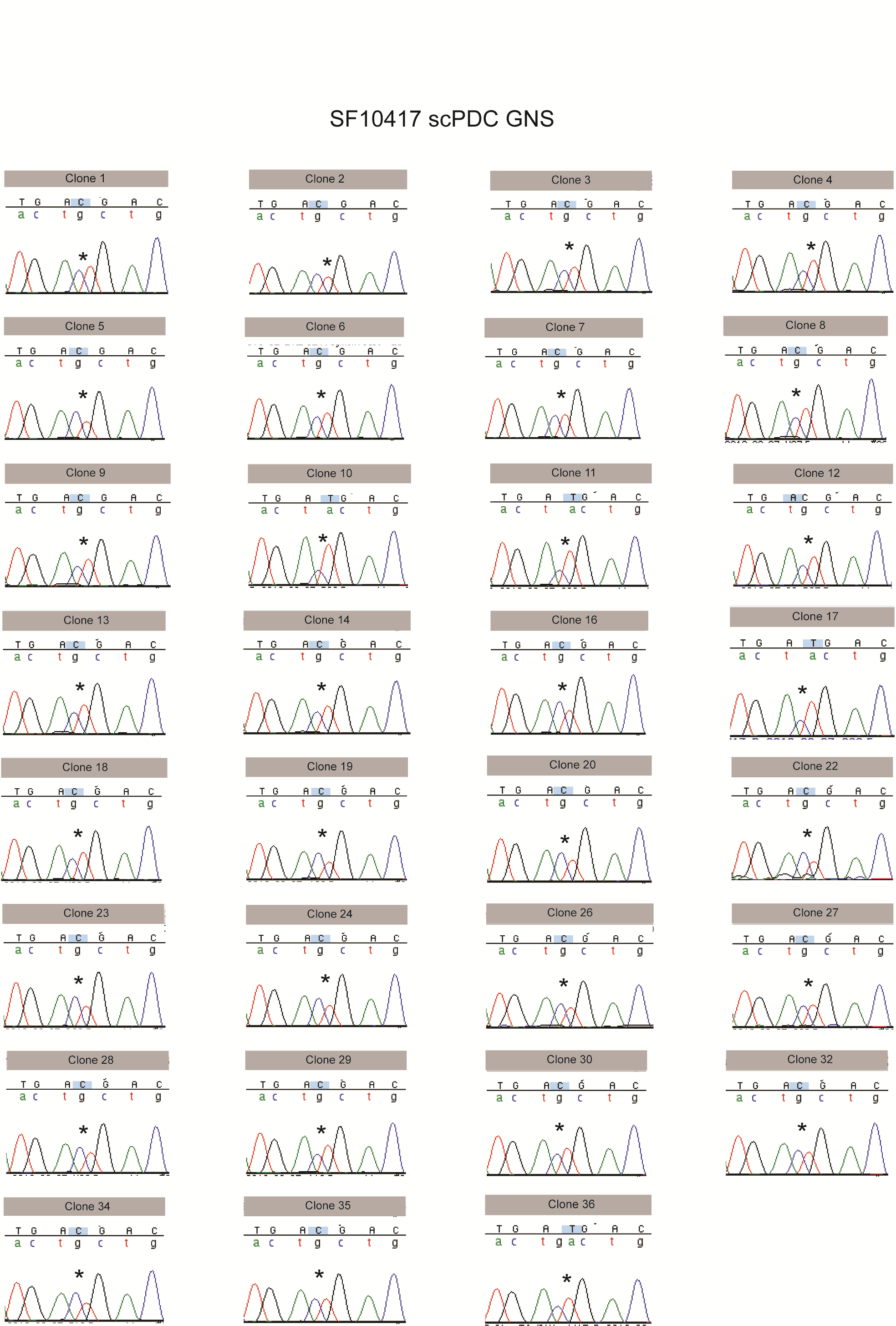

Supplement: vdaa088_suppl_Supplementary_Figure_S2 [file vdaa088_suppl_supplementary_figure_s2.png]

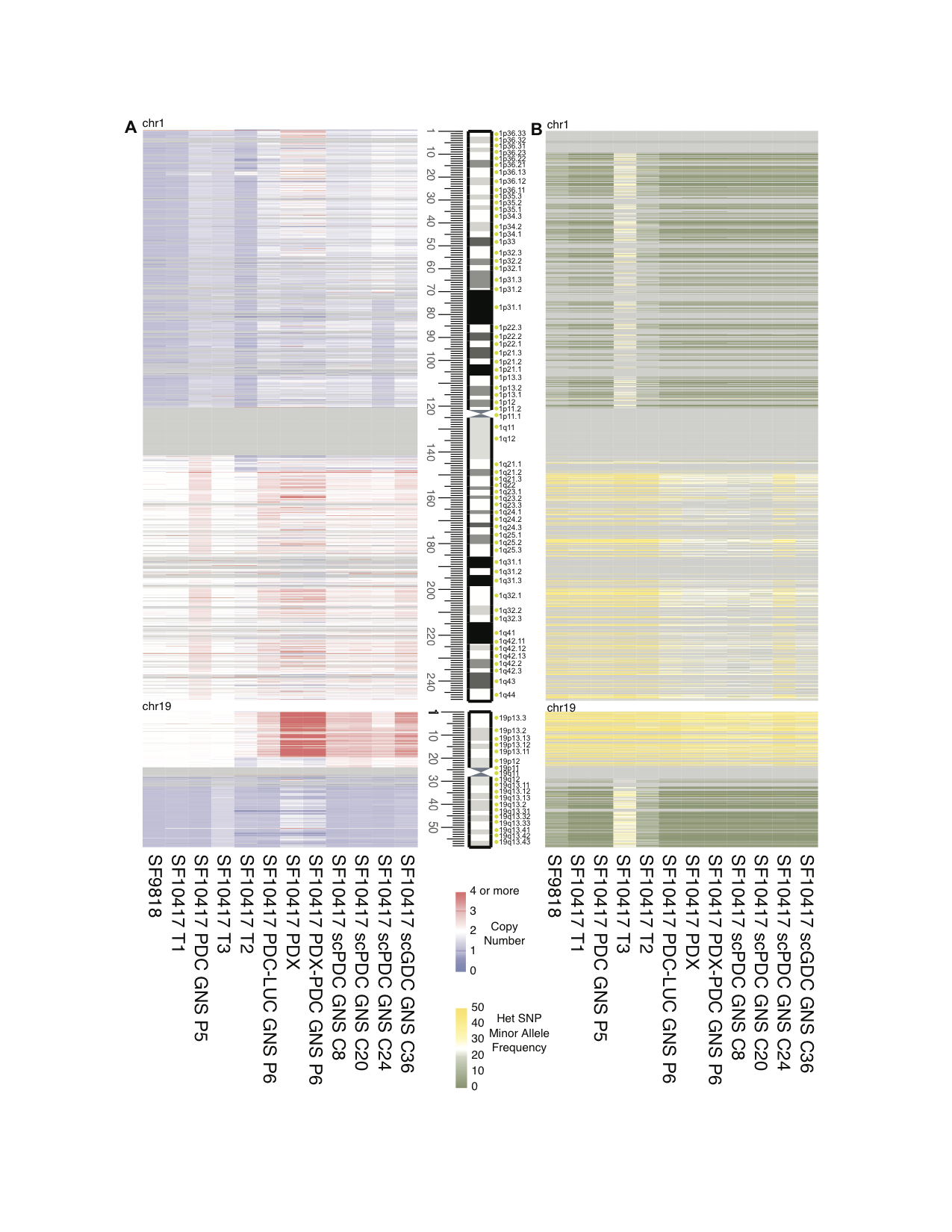

Supplement: vdaa088_suppl_Supplementary_Figure_S3 [file vdaa088_suppl_supplementary_figure_s3.png]

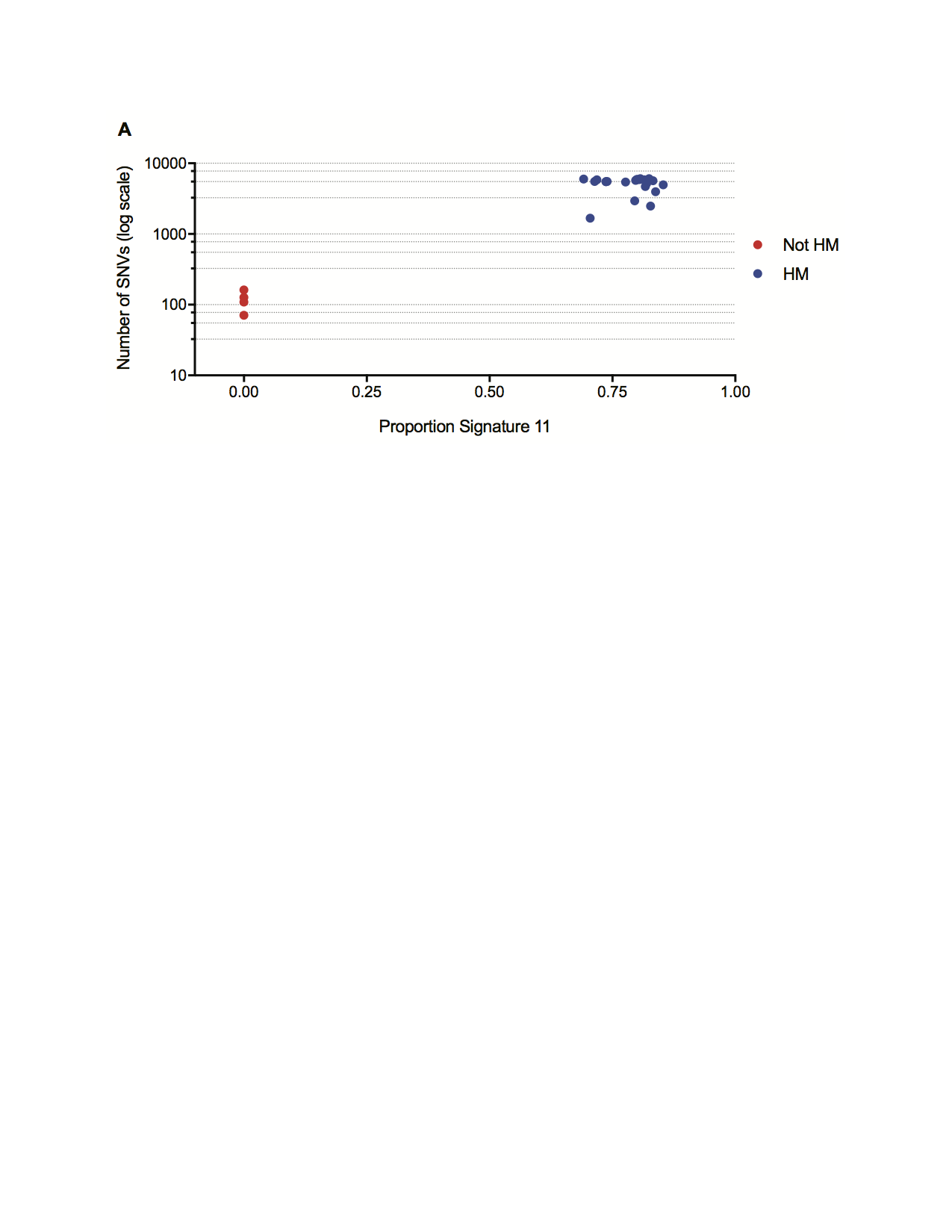

Supplement: vdaa088_suppl_Supplementary_Figure_S4 [file vdaa088_suppl_supplementary_figure_s4.png]
