# Supplementary material for: Patient-derived cells from recurrent tumors that model the evolution of IDH-mutant glioma
Source: Neurooncol Adv. 2020 Jul 16;2(1):vdaa088. doi: 10.1093/noajnl/vdaa088 (PMC7462278; doi:10.1093/noajnl/vdaa088)
Supplement: vdaa088_suppl_Supplementary_Legends [file vdaa088_suppl_supplementary_legends.docx]

**Supplementary Table 1.** Mean bait coverage for exome sequencing samples.

**Supplementary Table 2.** Mutation calls inferred from exome sequencing data for astrocytoma patient 137.

**Supplementary Table 3.** Mutation calls inferred from exome sequencing data for oligodendroglioma patient 278.

**Supplementary Figure 1. Astrocytoma scPDC maintain heterozygous IDH1 R132H.**

**Supplementary Figure 2. Oligodendroglioma scPDC maintain heterozygous IDH1 R132H.**

**Supplementary Figure 3. Copy number and LOH analysis of patient 278 shows derivation from a 1p/19q co-deleted parent line, with full 1p/19q LOH, including chromosomal regions evolving to copy neutral LOH, across all derived samples. (A)**, copy number along chromosome 1 and 19. **(B),**frequency of the minor allele for heterozygous SNPs along chromosome 1 and 19, in which 0 indicates full LOH (complete loss of the minor allele) and 50 indicates no LOH (a 50:50 ratio of minor:major alleles).

**Supplementary Figure 4. The mutation spectra of HM samples are dominated by TMZ-associated signature 11.** **(A)**, Exome data from all patient 137 and patient 278 tumor samples and PDC were analyzed for the contribution of known mutational signatures (Alexandrov, 2013). HM samples have a high mutation burden, and a high proportion of mutational signature 11, a signature associated with TMZ treatment.
